# Clinical and Cost-Effectiveness of Personalized Tele-Based Coaching for Farmers, Foresters and Gardeners to Prevent Depression: Study Protocol of an 18-Month Follow-Up Pragmatic Randomized Controlled Trial (TEC-A)

**DOI:** 10.3389/fpsyt.2020.00125

**Published:** 2020-03-04

**Authors:** Janika Thielecke, Claudia Buntrock, Ingrid Titzler, Lina Braun, Johanna Freund, Matthias Berking, Harald Baumeister, David D. Ebert

**Affiliations:** ^1^Department of Clinical Psychology and Psychotherapy, Institute of Psychology, Friedrich-Alexander-University Erlangen-Nürnberg, Erlangen, Germany; ^2^Department of Clinical Psychology and Psychotherapy, Institute of Psychology and Education, Ulm University, Ulm, Germany; ^3^Department of Clinical, Neuro- & Developmental Psychology, Vrije Universiteit Amsterdam, Amsterdam, Netherlands; ^4^GET.ON Institute, Hamburg, Germany

**Keywords:** prevention, depression, tele-based coaching, RCT, farmers

## Abstract

**Background:** Farmers show high levels of depressive symptoms and mental health problems in various studies. This study is part of a nationwide prevention project carried out by a German social insurance company for farmers, foresters, and gardeners (SVLFG) to implement internet- and tele-based services among others to improve mental health in this population. The aim of the present study is to evaluate the (cost-)effectiveness of personalized tele-based coaching for reducing depressive symptom severity and preventing the onset of clinical depression, compared to enhanced treatment as usual.

**Methods:** In a two-armed, pragmatic randomized controlled trial (*N* = 312) with follow-ups at post-treatment (6 months), 12 and 18 months, insured farmers, foresters, and gardeners, collaborating family members and pensioners with elevated depressive symptoms (PHQ-9 ≥ 5) will be randomly allocated to personalized tele-based coaching or enhanced treatment as usual. The coaching is provided by psychologists and consists of up to 34 tele-based sessions for 25–50 min delivered over 6 months. Primary outcome is depressive symptom severity at post-treatment. Secondary outcomes include depression onset, anxiety, stress, and quality of life. A health-economic evaluation will be conducted from a societal perspective.

**Discussion:** This study is the first pragmatic randomized controlled trial evaluating the (cost-)effectiveness of a nationwide tele-based preventive service for farmers. If proven effective, the implementation of personalized tele-based coaching has the potential to reduce disease burden and health care costs both at an individual and societal level.

**Clinical Trial Registration:** German Clinical Trial Registration: DRKS00015655.

## Introduction

Major depressive disorder (MDD) is a highly prevalent condition with lifetime prevalence rates estimated between 10.6 and 19.8% (1). Moreover, MDD is associated with substantial individual and societal burden due to functional impairment (2), increased mortality (3) as well as high socioeconomic costs (4, 5).

Despite the availability of effective treatments like psychotherapy and psychopharmacological interventions (6, 7), depression remains under-recognized and under-treated in primary care (8). Moreover, even in a scenario of full coverage of and compliance to evidence-based treatments, estimates suggest that only approximately one-third of the total MDD-related disease burden could be averted (9, 10). Therefore, prevention of MDD evokes global interest (11). Recent meta-analytic evidence suggests that psychological interventions could prevent or at least delay the onset of MDD by targeting individuals at elevated risk (e.g., selective prevention) or individuals with subclinical depression (e.g., indicated prevention) (12).

A large population-based study on anxiety and depression showed that male farmers and workers in related occupations had the highest level of depression of all 10 occupational groups of the Classification of Occupations (e.g., armed forces, legislators/managers, professionals including teachers, technicians, craft, and trade workers) (13). The Norwegian HUNT study found a doubled risk for farm-workers compared to their non-farming siblings to report depressive symptoms (14). Risk factors for depression associated with working in agriculture include: work-related stress (15, 16), additional jobs off the farm (15) and financial instability (17) paired with attitudinal barriers for help-seeking especially in male farmers (18, 19). Considering these risk factors, effective, and accessible preventive measures for these individuals are highly warranted and have the potential to greatly improve the well-being of this vulnerable group.

Face-to-face as well as internet-based psychological interventions have been shown to be effective at treating subthreshold depression and in preventing MDD onset (12, 20–22). However, both delivery modes possess certain limitations [e.g., low participation rates (23) or less use of online services in general and more adverse attitudes toward internet use (24)]. Even though access to the internet has greatly increased in Germany over recent years, rural areas still struggle with regards to the use of internet services (24, 25). Therefore, tele-based coaching might be a feasible adjunct to existing preventive interventions for depression, especially in rural communities.

Additionally, some evidence suggests that tele-based coaching is, compared to treatment-as-usual, effective in reducing depressive symptom severity in mildly to moderately depressed individuals with between-group effect sizes ranging from 0.60 at a 4-month follow-up in an intention-to-treat sample (26) to 0.44 at 12-month follow-up based on study completer-only data (27). However, the clinical and cost-effectiveness of tele-based coaching in the prevention of MDD remains understudied.

Thus, the present trial will evaluate whether personalized tele-based coaching is (cost-)effective in reducing depressive symptom severity and preventing the onset of clinical depression. This study is embedded in an evaluation project of a nationwide rollout of preventive services called “With us in balance,” carried out by the German social insurance for farmers, foresters, and gardeners (www.svlfg.de). The project aims to implement personalized tele-based coaching and internet-based interventions in conjunction with established, on-site prevention group workshops aimed to improve mental health amongst farmers, foresters, and gardeners.

The following research questions will be investigated in this pragmatic randomized controlled trial:

- Is personalized tele-based coaching effective in reducing depressive symptom severity compared to enhanced treatment as usual (TAU+) at post-treatment (primary outcome) and follow-ups (secondary outcome)?
- Is personalized tele-based coaching effective in preventing the onset of major depressive episodes as compared to TAU+ at follow-up?
- Is personalized tele-based coaching effective in reducing the severity of various mental health outcomes (e.g., stress, anxiety, insomnia) compared to TAU+?
- Is personalized tele-based coaching preferable to TAU+ in terms of costs and utilities in reducing depressive symptom severity?
- Which variables moderate and mediate the effects of personalized tele-based coaching on mental health outcomes?
- What is the level of satisfaction with, adherence to and acceptance of personalized tele-based coaching?
- Are there reported negative effects associated with personalized tele-based coaching?

## Methods and Analysis

### Study Design

The study is designed as a two-armed pragmatic randomized controlled trial comparing the clinical and cost-effectiveness of a personalized tele-based coaching provided by IVPNetworks (intervention group, IG) to a control group (CG) receiving enhanced treatment-as-usual (enhanced = e-mailed psychoeducation on stress, depression and information about access to regular care, TAU+). Assessments will take place at baseline (T0), post-treatment (6-month, T1) and at follow-ups at 12-month (T2) and 18-month (T3) after enrolment.

This clinical trial has been approved by the Medical Ethics Committee of the Friedrich-Alexander University Erlangen-Nürnberg (No. 345_18 B) and is registered in the German clinical trial register under DRKS00014000. The results will be reported in accordance with the Consolidated Standards of Reporting Trials (CONSORT) 2010 Statement and extension for reporting pragmatic trials (28–30).

### Participants and Procedure

#### Inclusion and Exclusion Criteria

##### Study participants

We will include farmers, foresters, and gardeners in Germany who (a) are insured by the SVLFG, (b) are an entrepreneur, collaborating spouse, assisting family member or pensioner, (c) are 18 years or older, (d) show elevated symptoms of depression (PHQ-9 ≥ 5), (e) have internet (for online assessments) and telephone access (for coaching), and (f) are willing to provide informed consent. Persons will be excluded if they (a) currently receive psychotherapy or (b) are not willing to sign a non-suicide contract in case of suicidal ideation as indicated by a score of one or greater on the Beck Depression Inventory (BDI-II) item 9 (“I feel I would be better off dead”) or a PHQ-9 total score of 20 or greater, or (c) primarily live in the German Federal States Bavaria or Schleswig-Holstein, since national roll-out into routine care started in these two states. As this is a pragmatic trial, we did not exclude comorbidities and current medication usage.

##### IVPNetwork coaches

Written informed consent to participate in online assessments will be obtained from coaches assigned to study participants. There are no specific eligibility criteria for participating coaches. Coaches are either self-employed or employed by IVPNetworks.

#### Recruitment

**Figure 1:**
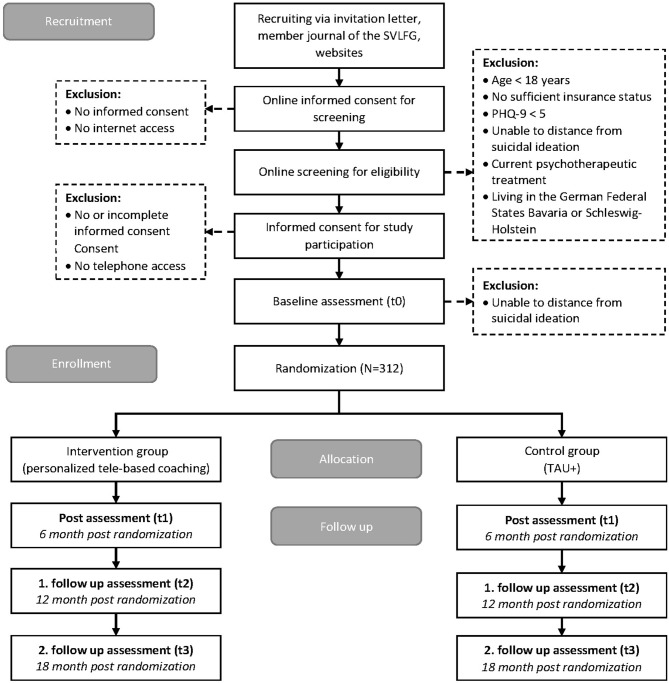
Overview of study procedure.

Invitation letters including information about the study procedures are sent to 120,000 randomly selected policyholders via postal mailings by the insurance company. The study is also listed on associated websites and in the member journal of the SVLFG (reaching 1.3 million policyholders). Interested individuals can respond to the study invitation via mail, e-mail, fax, and/or telephone, or can directly access the screening assessment through a URL or QR-Code. The study flow is visualized in Figure 1.

### Assessment of Eligibility and Randomization

Study eligibility is assessed with an online screening questionnaire. Persons who fulfill the inclusion criteria will receive detailed study information by e-mail along in addition to information on data security and their right to withdraw from the study at any time. After providing informed consent, participants will be invited to complete the baseline assessment (T0). If any of the inclusion criteria are not fulfilled but the person has a valid insurance status, the person will be redirected to the SVLFG call center for preventive services.

Randomization will take place at an individual level. Participants will be randomly allocated to the intervention or control condition consecutively after completing baseline assessment. A randomization list was created before the start of the study with the web-based program Sealed Envelope (www.sealedenvelope.com) by using permuted block randomization with randomly arranged block sizes (4, 6, 8, 12) and an allocation ratio of 1:1. A person not otherwise involved in the study will independently carry out the randomization procedure. Study participants cannot be blinded in this trial design but data collectors will be blinded to group allocation. The coaches providing the tele-based coaching are aware of treatment allocation; however, the coaches are not otherwise involved in the study. Participants will be informed about group allocation via e-mail.

### Intervention

All participants in the study will have unrestricted access to treatment-as-usual (e.g., general practitioner). The German S3-Guideline/National Disease Management Guideline Unipolar Depression (31) recommends a stepped care model in which more intensive treatments (i.e., cognitive behavioral therapy, antidepressant medication) are only provided if depressive symptoms intensify (i.e., diagnosed major depressive disorder). TAU was not protocolized in this pragmatic trial but will be monitored with the TiC-P (see Assessments) in order to provide an accurate description of the TAU utilized.

#### Intervention Condition

The personalized tele-based coaching is offered by IVPNetworks, which is a provider of integrated care in Germany. As evaluator, the study team has no influence on the design and content of the intervention. Even though the tele-based intervention does not focus on depression specifically but more general on mental health problems, it will be evaluated as part of a depression prevention program.

Participants assigned to the intervention group are registered with their contact details on the IVPNetworks management and documentation platform (IVPnet 2.0., www.ivpnet.de). A case manager assigns participants to their personal coach before the coach schedules the first session. The coaching consists of three major phases. (1) A beginning phase in which the first coaching session serves as an assessment of the participants' individual situation and provides time for mutual goal setting for the coaching. The individual goals guide the coaching process and are used to monitor participants' progress. Furthermore, building of a working alliance is emphasized in this first phase. (2) In the working phase, the coaching is intended to support participants in recognizing and understanding conflict patterns so that they can effectively cope with them by activating their own resources. Thus, the coaching focuses on the individual participant's personal situation and stressors (e.g., financial burden, family problems, work-related stress). (3) In the final phase of the coaching, the focus is on transferring learnt skills to everyday life and if needed further supporting offers (e.g., support groups or on-site group offers) are discussed and initiated to maintain coaching effects.

Personalized coaching implies that there are no fixed procedures or standardized manuals for the individual coaching process. Methods used during the coaching vary depending on the coach's therapeutic background. Coaches are qualified psychologists with at least a master's degree in psychology and completed a training in cognitive behavioral therapy, systemic therapy, psychodynamic therapy, hypnotherapy or other therapeutic trainings. All coaches, presumably 20–30, are managed by IVPNetworks and are either self-employed or employed by IVPNetworks. Licensed psychotherapists are available for supervision at any time. Furthermore, prior to interacting with any study participant, the coaches receive an introduction into common problems faced by farmers, foresters, and gardeners. The tele-based coaching intervention comprises a maximum of 34 sessions lasting for 25 or 50 min each over a 6 months period (maximum 850 min in total). The coaching duration and frequency are adapted to the participants' needs based on the individual coach's assessment. Theoretically, the coaching can be prolonged up to 9 months (resulting in up to an additional 150 min) after a new needs assessment has been done. If indicated, participants are supported in finding on-site social and health care services to complement the tele-based coaching (e.g., socioeconomic consultants, agricultural family counseling).

Alternatively, on-site coaching can be arranged if a participant no longer prefers tele-based coaching. In the case that a participant's depressive symptoms worsen at any time during the coaching, the participant will be stabilized and further supporting steps are arranged.

#### Control Condition

Participants in the control group (enhanced treatment as usual, TAU+) will receive psychoeducation materials via e-mail. Moreover, they will receive a link to an audiobook on how to deal with stress as well as a link to a platform for preventive services (https://17358.zentrale-pruefstelle-praevention.de/kurse/) in routine care in order to facilitate access to service use.

### Assessments

Assessments at screening, baseline (T0), post-treatment (T1), and follow-up (T2–T3) will be conducted via a secured online platform (www.unipark.de). For an overview of instruments, see Table 1. Participants in both study conditions will receive €15 for each completed post-treatment or follow-up assessment as an incentive to continue study participation, resulting in a total of €45 for study completion.

**Table 1:** Overview of the assessments.

**Instruments**	**Description**	**Time of measurement**				
		**Screening**	**T0**	**T1**	**T2**	**T3**
**Screening instruments**						
PHQ-9	Patient Health Questionnaire	x				
**Primary outcome**						
QIDS-SR16	Quick Inventory for Depressive Symptomatology		x	x^a^	x	x
**Secondary outcomes**						
Adapted items from CIDI 3.0, CIDI-SC, and Epi-Q Screening Survey	Prevalence of major depression		x	x	x	x
Adapted items from CIDI 3.0, CIDI-SC, and Epi-Q Screening Survey	Prevalence of bipolar disorder and anxiety disorder		x			
PSS-10	Perceived Stress Scale		x	x	x	x
ISI	Insomnia Severity Index		x	x	x	x
SSS-8	Somatic Symptom Scale		x	x	x	x
GAD-7	Generalized Anxiety Disorder		x	x	x	x
PAS	Panic and Agoraphobia Scale		x	x	x	x
AUDIT-C	Alcohol use disorder identification test—Consumption questions		x	x	x	x
MBI-GS	Maslach-Burnout-Inventory		x	x	x	x
AQoL-8D	Quality of life		x	x	x	x
SPE	Subjective Prognosis of Gainful Employment Scale		x	x	x	x
**Cost measurement**						
TiC-P	Utilization of health services, work-related productivity		x	x	x	x
**Intervention-related outcomes**						
WAI-SR^b^, WAI-SRT^c^	Therapeutic relationship			x	x	
adapted CSQ-I	Patient satisfaction			x	x	
INEP^b^	Inventory of negative effects in psychotherapy			x	x	
Negative effects (open questions)^b^	Negative effects in psychotherapy			x	x	
**Other assessments**						
Socio-demographics	Sex, age, farm type, family situation		x			
Predictors	Predictors for development of major depression and anxiety		x			
BDI-II	Suicidality-Item	x	x	x	x	x
Reporting data IVPNetworks	Adherence, drop-outs, Coaching length, amount of sessions, topics (keywords)		x	x		

a *Primary outcome is the standardized mean difference between intervention and control group at T1. QIDS-SR16 will also be assessed at T0 and T2-T3*.

b *Recorded in intervention group only*.

c *Recorded in coaches only*.

*T0 Baseline, T1 6 months, T2 12 months, T3 18 months after randomization*.

#### Screening

The preliminary screening assesses sociodemographic variables (e.g., age, gender, residence, employment relationship, insurance status), current psychotherapy status and depressive symptomology to validate eligibility for participation.

Depressive symptomology is assessed using the German Version of the Patient Health Questionnaire (PHQ-9) (32). The screening inventory consists of nine items on a 4-point-scale with a rating scale ranging from 0 to 3 (0 = “not at all,” 1 = “several days,” 2 = “more than half the days,” 3 = “nearly every day”) with each item assessing one symptom criterion domain of MDD. Total score ranges from 0 to 27 with higher scores indicating more severe symptoms. Scores from 0 to 4, 5 to 9, 10 to 14, 15 to 19, and 20 to 27 indicate minimal, mild, moderate, moderately severe, and severe depression severity, respectively (33). An additional item assesses severity of daily life limitations associated with depressive symptoms. The computerized version of the PHQ-9 (α = 0.88) and the paper-pencil version (α = 0.89) show equally high internal consistency (34).

Additionally, the suicide item of BDI-II (35) will be applied to screen for suicidal ideation if the suicidal item of PHQ-9 is greater than or equal to one. Participant's contact information is recorded in order to monitor suicidal ideation independent of eligibility for study participation. Lastly, to better understand recruitment tactics, participants are asked which recruitment activity made them aware of the study.

#### Outcome Measurements

##### Primary outcom

*Depressive symptom severity at post-treatment (T1)*. Depressive symptom severity (primary outcome) at post-treatment (T1), is assessed with the German Version of the Quick Inventory Depressive Symptomology (QIDS-SR16) (36). With 16 items (4-point-scale ranging between 0 and 3), the self-report inventory covers all nine DSM-5 symptom criterion domains of MDD. When analyzing scores, only the highest rated item for sleep, weight, and psychomotor activity is included leading to total scores ranging between 0 and 27, with a higher score indicating more depressive symptom severity. Scores between 0 and 5, 6 and 10, 11 and 15, 16 and 20, and >20, indicate normal health status, or mild, moderate, severe, or very severe depressive symptom severity, respectively (36). Compared to current and lifetime diagnosis based on Structured Clinical Interview for DSM-IV-TR Axis I Disorders (SCID), the QIDS-SR16 proved to be a reliable screening instrument for a diagnosis of clinical depression (37) with good in (α = 0.86) (36).

##### Secondary outcomes

*Depressive symptom severity at follow-up (T2 - T3)*. Additionally, depressive symptom severity at follow-up assessments at 12-months (T2) and 18-months (T3) will be measured with the QIDS-SR16.

*Onset of ICD-10 major depressive episode and bipolar disorder*. The onset of major depressive episodes and bipolar disorder, are assessed with adapted items from the web version of the Composite International Diagnosis Interview version 3.0 (CIDI 3.0) and Screening Scales (CIDI-SC) (38) and the Epi-Q Screening Survey (39) as used in the WHO World Mental Health Surveys International College Student Project (40). Bipolar disorder will be assessed at baseline (T0), while depressive episodes will be assessed at all time points (T0–T3).

In addition, the QIDS-SR16 is used to identify possible clinically relevant cases of depression. A total score of 13 and greater is defined as cut-off for possible cases of clinical depression for all time points (T0–T3). This value was selected as it yield best results for sensitivity (76.5%) and specificity (81.8%) and resulted in a correct classification of over 80% of participants (37).

*Perceived stress*. Perceived stress is assessed with the perceived stress scale (PSS-10) in which ten items are rated on a 5-point Likert scale (41, 42). The German Version of the perceived stress scale assess perceived stress in the past week and is reported with good internal consistency (α = 0.84) (42, 43).

*Insomnia severity*. The Insomnia Severity Index (ISI) (44) is used to measure insomnia severity in participants. The questionnaire has been validated to identify cases of clinical insomnia. The ISI has adequate discriminative validity of the individual items and a high internal consistency (α = 0.90–0.92). The self-report questionnaire consists of seven items on a 5-point Likert scale (45, 46). Examination of the German version of the ISI in three cross-sectional studies in different target groups revealed acceptable to good internal consistency (α = 0.76–0.81) (47)

*Somatic symptom burden*. Somatic symptom burden will be assessed with the Somatic Symptom Scale, the short version of the PHQ-15 questionnaire (48). In 8 items (5-point Likert scale), eight symptoms common in primary care and relevant for mental health are assessed (gastrointestinal symptoms, pain, fatigue, cardiopulmonary symptoms). Additionally, a study in a German sample of participants reported good reliability (α = 0.81) and correlation with depression and other health outcomes (49).

*Generalized anxiety disorder*. The GAD-7 (50), a 7-item self-report questionnaire with a 4-point Likert scale, is used for screening and severity measuring of Generalized Anxiety Disorder (GAD). Previous studies have found that the internal consistency of this instrument is only slightly lower in a German sample (α = 0.89) (51) compared to a sample in the United States (α = 0.92) (50). The GAD-7 is considered a reliable screening instrument for GAD (50, 51)

Additionally, the presence of GAD at baseline is assessed with adapted items from the Composite International Diagnosis Interview version 3.0 (CIDI 3.0), Screening Scales (CIDI-SC) (38) and the Epi-Q Screening Survey (39).

*Panic and agoraphobia scale*. The panic and agoraphobia scale (52, 53) will be assessed to measure severity of panic and agoraphobic symptoms.

Twelve items with a 5-point Likert scale assess five individual subscales: regarding panic attacks, agoraphobic avoidance, anticipatory anxiety, daily life limitations, and health concerns. An additional item (6-point Likert scale) differentiates between unexpectedness vs. expectedness of panic attacks and is not included in the calculation of the total score (52). Additionally, situations in which individuals believe panic attack are likely to occur are recorded. Good internal consistency is reported for the panic and agoraphobia scale (α = 0.88) (52).

*Alcohol consumption*. Alcohol consumption will be assessed with the Alcohol Use Disorder Identification Test-Consumption Questions (AUDIT-C) (54). As previous research has shown, the first three items of the AUDIT can be used as a reliable stand-alone screening of risky alcohol consumption (AUDIT-C) (55). Good internal consistency is reported for the German version of the AUDIT-C (α = 0.80) (56). In the present study, the German version of the AUDIT-C, as distributed by the German Medical Association (https://www.bundesaerztekammer.de/fileadmin/user_upload/downloads/AlkAUDITCFragebogen.pdf) is administered.

*Burnout*. The Maslach Burnout Inventory (57) is used to record burnout symptomology. The Inventory assess the three dimensions of burnout including “Emotional Exhaustion,” “Cynicism,” and “Professional Efficacy” with 22 items and a 7-point Likert scale. The reliability (α = 0.71–0.88 for subscales) has been proven acceptable in cross-national comparison (58)

*Quality of life*. The self-report questionnaire Assessment of Quality of Life (AQoL-8D) will be used to assess health-related quality of life. With 35 items (5-point Likert scale) three physical dimensions (“independent living,” “pain,” and “senses”) as well as the five psycho-social dimensions (“mental health,” “happiness,” “coping,” “relationships,” and “self-worth”) are assessed, yielding two distinct sum scores (59). The questionnaire is reported with excellent reliability (α = 0.96) and good psychometric properties (59).

*Subjective capacity to work*. The participants subjective capacity to work will be measured using the 3-item Subjective Prognosis of Gainful Employment Scale (60). The short self-report scale showed high internal consistency (Guttman scaling: rep = 0.99) and validity for assessment of subjective endangerment and prognosis of capacity to work (60).

*Cost measures*. For cost evaluation, the German version of the Dutch cost questionnaire “Trimbos Institute and Institute of Medical Technology Questionnaire for Costs Associated with Psychiatric Illness” (TiC-P) (61) was adapted to specifically assess farmers, foresters, and gardeners. The self-report questionnaire assesses direct medical costs (e.g., use of health care services, visits to the general practitioner, use of medications, sessions with psychotherapists or psychiatrists, inpatient hospital care), direct non-medical costs (e.g., patient and family costs), and indirect costs (e.g., productivity losses due to absenteeism and presenteeism). The German version has been widely used in health economic outcome evaluations alongside clinical trials (62–65).

##### Intervention-related outcomes

As part of the evaluation, the process and content of the tele-based coaching will be documented and reported in detail during the intervention phase and at the end of the coaching (e.g., amount of completed sessions and their duration, common topics in coaching sessions, compliance of participants, referrals to onsite services). The qualification of coaches will be assessed with a sociodemographic questionnaire. In addition, to gain deeper insights the coaching procedure and methods, interviews in a subsample of coaches will be conducted.

*Intervention satisfaction*. At post-treatment (T1) and the first follow-up (T2) participants' satisfaction with the personalized tele-based coaching is measured using a German Version of the Client Satisfaction Questionnaire (CSQ-8, adapted version for internet interventions: CSQ-I) (66–68). In the IG, the CSQ-I will be adapted for telephone coaching (the original CSQ-8 is validated only in face-to-face contexts). The CSQ-8 as well the CSQ-I consist of eight items (4-point Likert scale) and report a high internal consistency (α = 0.87 and 0.93) (67, 68). An adapted version of the CSQ-I will be used for the assessment of participant's satisfaction with the information material received in the CG.

*Working alliances*. The therapeutic alliance between participants and coaches will be addressed with the German short version of the Working Alliance Inventory (WAI-SR) (69). The three subscales “agreement on tasks,” “agreement on goals,” and “development of an affective bond” are assessed with 12 items on a 5-point Likert scale. The internal consistencies for the German version are between α = 0.81 and α = 0.91 for the subscales and between α = 0.90 and α = 0.93 for the total score (69, 70). The WAI-SR will be applied in the IG at T1 and T2, respectively. Furthermore, the coaches will be asked to complete the 10-item therapist version (WAI-SRT, developed by Adam O. Horvath, wai.profhorvath.com) at the same time. This approach provides the opportunity to evaluate the therapeutic relationship from two different points of view (i.e., the participant and the coach) and to report a differentiated and comprehensive picture of the experienced working alliance. The WAI-SR and the WAI-SRT were adapted for the current trial to assess coaching in a prevention setting. The items were adjusted to refer to “coaches” instead of therapists and to “telephone coaching” instead of therapy.

*Side effects of the intervention*. Side effects of the intervention will be assessed with a version of the Inventory for the Assessment of Negative Effects of Psychotherapy (71) adapted to the tele-based setting of the intervention. This 22-item questionnaire assesses whether any negative changes were experienced during or after the tele-based coaching that are causally attributed to the intervention. Furthermore, open-ended questions will be included for qualitative assessments of possible negative side effects of the personalized tele-based coaching. Symptom deterioration will be assessed using the Reliable Change Index based on QIDS at post-treatment and follow-ups.

##### Other assessments

*Covariates*. Variables that could potentially moderate the expected effects, including sociodemographic as well as information about the agricultural business (e.g., farm size, area cultivated, number of workers) and the overall family and work situation (e.g., financial situation, number of relatives living and working together, general work load), will be assessed at baseline (T0).

Furthermore, we will explore variables associated with depression (72–74) or that have shown to predict treatment outcome for depression and anxiety (75). Clinical characteristics that shall be investigated include depressive symptom severity (76), lifetime history of MDD or any mental disorder (77–80), past or present suicidal thoughts or plans (35, 79–81), experience with psychotherapy (82), treatment motivation (83), treatment preference (84–86), family history of mental illness (77, 87–89) (chronic) illness, self-perceived health and energy (61, 78, 90–95), traumatic or adverse childhood experience (abuse, parental death, or divorce) (80, 87, 88, 96–101), body satisfaction and eating disorder (94, 102–107), sleep quality (44, 90, 94, 108, 109), and accidents and injuries (15, 110).

Personality characteristics include smoking (82, 94, 111), alcohol consumption and drug use (77, 91, 94), relationship quality (91, 112, 113) (partner) violence experience (96, 112, 114–116), threatening life events in the past 12 months (79, 80, 91, 117–120), physical activity and sedentary behavior (94, 106, 111, 121–123), stress (15, 43, 79, 124, 125), need for affect, emotional avoidance (126–128), anxiety sensitivity (129–131), sense of mastery, internal locus of control (79, 132–135), worry (136–138), self-worth (100, 104, 139–141), and loneliness (142–145).

Sociodemographic variables that shall be examined include age (76, 77, 111, 146, 147), sex (82, 92, 104, 110, 111, 117, 118, 146–149), origin country (148), migration (150–153), ethnicity, minority (111, 147, 154), discrimination (103, 155, 156), education level (92, 96, 109–111, 148), employment status (88, 111, 125), relationship status, living situation (88, 92, 111, 146), parenthood (82), caregiving living situation (94, 157, 158), economic status (90, 91, 118, 159), and social status (94, 160, 161).

*Safety monitoring*. Suicidal ideation will be screened in all assessments using PHQ-9 (screening) or QIDS-SR16 (T0–T3). If the suicide item of PHQ-9 or QIDS-SR16 has a score of one or greater, the BDI-II (35) suicide item will additionally be assessed.

A score of one or greater on the BDI-II suicide item or a PHQ-9 score of 20 greater / QIDS-SR16 score of 16 or greater [indicating severe depressive symptoms (36)] will result in a standardized suicide protocol. Participants will receive e-mails with detailed information on 24/7 health services and are advised to seek professional help if symptoms persist or increase. The wording of this information is adapted to the severity of the indicated suicidality as given in the BDI-II Item or PHQ-9/QIDS-16 scores. In cases of a BDI-II scores of 2 or 3, and/or severe depressive symptoms, the persons are called by a psychologist or a psychotherapist (licensed or in training) within 3 days for evaluation of suicidal ideation severity and dissociation. The suicide protocol is developed and closely monitored by a licensed psychotherapist.

As part of the suicide protocol, at screening and baseline, all participants with suicidal ideation and/or severe depressive symptoms are asked to return a signed non-suicide contract if they are able to distance themselves from suicidal ideation (inclusion criteria). Participants are reminded that the coaching is not designed for persisting suicidal ideation and are provided necessary resources (as discussed previously).

### Statistical Analyses

#### Sample Size/Power Calculation

The estimated effect size of d = 0.35 used for this study is based on the findings of the meta-analysis by Cuijpers et al. (20) regarding the effect of psychological interventions on subclinical depression (*g* = 0.35, 95%-CI: 0.23–0.47) and effect sizes commonly observed in studies on health outcomes (162).

Power calculation conducted with G^*^Power (Version 3.1.9.2) for a two-sided *T*-Test (α = 0.05, β = 0.80, d = 0.35) resulted in *N* = 260. In order to account for dropouts during the 18-month follow-up, we calculated an extra 20% of participants, taking into account reported dropout rates between 20 and 23% from studies with telephone coaching in different settings (27, 163). Accounting for drop-outs resulted in the final *N* = 312.

#### Clinical Analyses

Clinical analysis will be performed using R statistic software (164). All statistical analyses will be performed based on intention-to-treat principle. Missing data will be imputed using a Markov Chain Monte Carlo multivariate imputation algorithm. Group difference between pre- (T0) and post-assessment (T1) in the primary outcome will be analyzed using generalized linear modeling adjusting for baseline depressive symptom severity. Within- and between-group Cohen's d effect sizes [and their 95% CIs according to Hedges and Olkin (165)] controlling for baseline data (i.e., calculating change scores divided by the pooled standard deviation of change scores) will be reported. Improvements in the primary outcome at individual level will be examined by assessing the number of participants who displayed a treatment response defined by the Reliable Change Index as proposed by Jacobson and Truax (166) and a close-to-symptom-free status (e.g., QIDS ≤ 5). Close-to-symptom-free status will only be evaluated in the subgroup of participants who reported at least mild depressive symptom severity at baseline. Group differences in depression onset will be assessed using Poisson regression models in the subset of participants who did not meet the diagnostic depression status at baseline as assessed with the self-report web version of the adapted CIDI. Group differences in diagnostic status in the subset of participants who did not meet depression diagnosis criteria at baseline will be analyzed using logistic regression models. In sensitivity analyses, we will assess the influence of objective measures, such as therapeutic background of coaches and length of coaching, on intervention outcomes. Other secondary outcomes like anxiety, perceived stress, sleep quality, and quality of life will be analyzed as the primary outcome. Additionally, moderation and mediation analyses will be performed.

#### Economic Evaluation

We will conduct and report the health economic evaluation in agreement with the Consolidated Health Economic Evaluation Reporting Standards (CHEERS) statement (167) and the guidelines from the International Society for Pharmacoeconomics and Outcomes Research (ISPOR) (168). The health-economic evaluation will involve a combination of a cost-effectiveness analysis and a cost-utility analysis (169). The economic evaluation will be performed from a societal perspective and from the perspective of the social insurance company with a time horizon of 18 months. In the cost-effectiveness analysis, the incremental cost-effectiveness ratio (ICER) will be calculated by dividing incremental costs (e.g., cumulative per-participant costs) by the unit of effect gained (e.g., reliably improved case or quality-adjusted life years based on the AQoL-8D). The corresponding equation is ICER = (CostsIG – CostsCG) / (EffectsIG – EffectsCG), with IG representing the intervention group and CG the control group (169). To handle sampling uncertainty, we will bootstrap the seemingly unrelated regression equations model (SURE model) to generate 2,500 simulations of cost and effect pairs while allowing for correlated residuals of the cost and effect equations and adjusting for potential confounding variables (170). Based on the bootstrapped SURE model, bias-corrected and accelerated 95% confidence intervals will be obtained for incremental costs and effects while 95% confidence intervals around ICERs will be obtained by the bootstrap acceptability method as proposed by Glick et al. (171). The bootstrapped cost and effect pairs will be plotted in a cost-effectiveness plane. The cost-effectiveness plane is used to visually represent the differences in costs and health outcomes between the tele-based coaching and TAU+ in two dimensions. Health outcomes (effects) are plotted on the x-axis and costs on the y-axis. TAU+ is plotted at the origin, and so the x and y values represent incremental health outcomes and incremental costs vs. routine care. The cost-effectiveness plane is divided into four quadrants: the north-east (NE) quadrant, in which new interventions generate more health gains but at higher costs; the south-east (SE) quadrant, in which new interventions costs less and create higher health effects (e.g., the new intervention dominates the alternative); the north-west (NW) quadrant, in which new interventions produce less health effects at higher costs (e.g., the new intervention is dominated by the alternative) and the south-west (SW) quadrant, in which new interventions costs less but also generate less health effects (169). To disclose the probability that the intervention is cost-effective for a range of willingness-to-pay ceilings (e.g., the maximum a decision maker is willing to pay for a unit of effect), the bootstrapped cost and effect pairs will also be shown in a cost-effective acceptability curve (172). To test the robustness of the base-case findings, multi-way sensitivity analyses will be completed. The analysis will be performed using StataCorp (173).

## Discussion

This study will be the first pragmatic randomized controlled trial to examine the clinical and cost-effectiveness of personalized tele-based coaching for farmers, foresters, and gardeners in Germany. The intervention is meant to prevent clinical depression and reduce depressive symptom severity in farmers, foresters, and gardeners.

The study is embedded in a large scale nationwide prevention project which does not only aim at the evaluation of the (cost-)effectiveness but also target nation-wide implementation at the same time. Stepwise national rollout of the intervention runs parallel to this randomized controlled trial. Confounding is controlled for via recruitment activities and eligibility criteria. Therefore, if proven effective, the personalized tele-based coaching will be sustainably introduced into regular care in Germany, which can be seen as a strength of this trial since most studies in the field of depression prevention lack a sustainable integration in the health care system (174, 175). Study findings can therefore directly contribute to improving the preventive service of the SVLFG.

Another major strength of tele-based coaching compared to face-to-face psychological interventions or mental health workshops is that delivery over the telephone greatly reduces the time and effort associated with people affected in the rural area (e.g., no travel time to visit a therapist).

Some limitations of the study should also be mentioned. First, the assumed advantage of tele-based coaching compared to internet-based interventions that telephone coaching does not rely on internet access and might therefore be more accessible in rural areas, cannot be verified with this study. Participants are only enrolled in the study if they are able to complete the online-based assessments and therefore require internet access for study participation. Nevertheless, participants in IG and CG will be asked about their preferences for treatment of mental health problems (e.g., face-to face, online, telephone, combined delivery modes) in order to get first insights in setting preferences.

Second, the operationalization of the personalized tele-based coaching intervention itself is difficult. As Ammentorp et al. (176) claims, detailed descriptions of coaching interventions and methods used are crucial in order to improve the field of life coaching research and its impact on (mental) health outcomes. Although this intervention is highly personalized with regard to contact duration, frequency, and covered coaching topics, we will be able to give a detailed and comprehensive description of the intervention by routine data documentation in addition to data collected from coaches and participants. Thereby, we will add to this growing field of research by providing strongly needed evidence from a randomized controlled trial regarding preventive coaching interventions. The information gained could also be used in the development of standardized coaching manuals.

Third, as mentioned earlier, previous research does not provide profound evidence about estimates regarding effect sizes and drop-out rates. We accounted for possible drop out by increasing the target sample by 20%. Nevertheless, we are at a risk to over- or underestimate dropout. Reporting effect sizes and drop-out rates will help researchers calculate target sample sizes in future studies.

To sum up, this pragmatic trial leads to a robust estimation of the (cost-)effectiveness of personalized tele-based coaching in the target population. Therefore, results from the study can be generalized to farmers, foresters, and gardeners in Germany and comparable coaching situations.

## Ethics Statement

The studies involving human participants were reviewed and approved by Ethics Committee of the Friedrich-Alexander-University Erlangen-Nürnberg (no. 345_18 B). The patients/participants provided their written informed consent to participate in this study.

## Trial Status

The recruitment of participants started in December 2018 and has been finished in April 2019 while the first draft of this paper was composed. Data collection is still ongoing and no analyses have been performed, yet.

## Author Contributions

DE, HB, and MB obtained funding for this study. DE, IT, CB, JT, and HB contributed to the study design. CB contributed to the design of the economic evaluation study. JT drafted the manuscript, supervised by CB, and is responsible for recruitment and coordination of the trial. JF and LB contributed to the acquisition of data and managing of recruitment. IT and CB are supervising the recruitment and trial management. All authors provided critical revision of the article and approved the final manuscript.

### Conflict of Interest

DE has served as a consultant to/on the scientific advisory boards of Sanofi, Novartis, Minddistrict, Lantern, Schoen Kliniken, Ideamed, and German health insurance companies (BARMER, Techniker Krankenkasse) and a number of federal chambers for psychotherapy. DE and MB are stakeholders of the Institute for health training online (GET.ON), which aims to implement scientific findings related to digital health interventions into routine care. HB reports to have received consultancy fees and fees for lectures/workshops from chambers of psychotherapists and training institutes for psychotherapists in the e-mental-health context. IT reports to have received fees for lectures/workshops in the e-mental-health context from training institutes for psychotherapists. She is implementation lead and project lead for the EU-project ImpleMent. All at the Institute for health training online (GET.ON). The remaining authors declare that the research was conducted in the absence of any commercial or financial relationships that could be construed as a potential conflict of interest.

## Abbreviations

AQoL-8D: Assessment of Quality of Life
AUDIT: Alcohol Use Disorder Identification Test—Consumption Questions
BDI-II: Beck Depression Inventory
CG: control group
CIDI: Composite International Diagnosis Interview
CSQ: Client Satisfaction Questionnaire
GAD: Generalized Anxiety Disorder
ICER: Incremental cost-effectiveness ratio
IG: intervention group
ISI: Insomnia Severity Index
MDD: Major depressive disorder
PHQ: Patient Health Questionnaire
QIDS-SR16: Quick Inventory Depressive Symptomology
SVLFG: Social Insurance for Agriculture, Forestry and Horticulture
TAU: Treatment as usual
TiC-P: Trimbos Institute and Institute of Medical Technology Questionnaire for Costs Associated with Psychiatric Illness
WAI: Working Alliance Inventory.
